# Wearable Inertial Measurement Units for Assessing Gait in Real-World Environments

**DOI:** 10.3389/fphys.2020.00090

**Published:** 2020-02-20

**Authors:** David Renggli, Christina Graf, Nikolaos Tachatos, Navrag Singh, Mirko Meboldt, William R. Taylor, Lennart Stieglitz, Marianne Schmid Daners

**Affiliations:** ^1^Product Development Group Zurich, Department of Mechanical and Process Engineering, ETH Zurich, Zurich, Switzerland; ^2^Institute for Biomechanics, Department of Health Sciences and Technology, ETH Zurich, Zurich, Switzerland; ^3^Department of Neurosurgery, University Hospital Zurich, Zurich, Switzerland

**Keywords:** natural walking patterns, gait analysis, IMU sensors, ZurichMOVE, non-controlled settings, real-world environment, walking disorder, hydrocephalus

## Abstract

**Background:**

Walking patterns can provide important indications of a person’s health status and be beneficial in the early diagnosis of individuals with a potential walking disorder. For appropriate gait analysis, it is critical that natural functional walking characteristics are captured, rather than those experienced in artificial or observed settings. To better understand the extent to which setting influences gait patterns, and particularly whether observation plays a varying role on subjects of different ages, the current study investigates to what extent people walk differently in lab versus real-world environments and whether age dependencies exist.

**Methods:**

The walking patterns of 20 young and 20 elderly healthy subjects were recorded with five wearable inertial measurement units (ZurichMOVE sensors) attached to both ankles, both wrists and the chest. An automated detection process based on dynamic time warping was developed to efficiently identify the relevant sequences. From the ZurichMOVE recordings, 15 spatio-temporal gait parameters were extracted, analyzed and compared between motion patterns captured in a controlled lab environment (10 m walking test) and the non-controlled ecologically valid real-world environment (72 h recording) in both groups.

**Results:**

Several parameters (*Cluster A*) showed significant differences between the two environments for both groups, including an increased outward foot rotation, step width, number of steps per 180° turn, stance to swing ratio, and cycle time deviation in the real-world. A number of parameters (*Cluster B*) showed only significant differences between the two environments for elderly subjects, including a decreased gait velocity (*p* = 0.0072), decreased cadence (*p* = 0.0051) and increased cycle time (*p* = 0.0051) in real-world settings. Importantly, the real-world environment increased the differences in several parameters between the young and elderly groups.

**Conclusion:**

Elderly test subjects walked differently in controlled lab settings compared to their real-world environments, which indicates the need to better understand natural walking patterns under ecologically valid conditions before clinically relevant conclusions can be drawn on a subject’s functional status. Moreover, the greater inter-group differences in real-world environments seem promising regarding the sensitive identification of subjects with indications of a walking disorder.

## Introduction

Important indications of a person’s health status can be obtained through analysis of walking patterns (König et al., 2014; Ravi et al., 2019). A variety of neurological disorders show specific gait impairments such as dementia (Allan et al., 2005; McArdle et al., 2019), normal pressure hydrocephalus (NPH) (Stolze et al., 2000, 2001) or Parkinson’s disease (Stolze et al., 2001; Del Din et al., 2016). The diagnosis of these diseases is difficult, and misinterpretation is possible, especially in NPH. Here, difficulties arise in distinguishing the disease from other neurodegenerative diseases such as subcortical arteriosclerotic encephalopathy, polyneuropathy, or spinal canal stenosis (Bradley et al., 1991; Hebb and Cusimano, 2001; Relkin et al., 2005). Early indications suggest that subtle characteristics contained within a subject’s gait patterns are sufficient to differentiate neurological pathologies at an early time point, and could form a fundamental basis for aiding clinical decision making (König et al., 2016a, b). With sufficient objectivity, such information could therefore support the clinical diagnosis of e.g., NPH, where it is estimated that only one in ten cases is correctly diagnosed and correctly treated (Jaraj et al., 2014). According to literature, NPH exhibits specific gait characteristics such as increased foot outward rotation, increased number of steps needed for a 180° turnaround, increased cycle time deviation, and impaired arm swing compared to asymptomatic controls (Stolze et al., 2000, 2001; Relkin et al., 2005; Gallia et al., 2006; Shrinivasan et al., 2011).

Medical examination of walking patterns is typically performed in a hospital or doctor’s office environment by visual inspection. If a disease is suspected, patients are sent to specialized centers for further investigation. However, this examination process shows three main deficits: (1) Examinations in a doctor’s office are rather low in complexity and cover only a small range of walking pattern characteristics. Subtle characteristics within walking patterns are often not visible to the naked eye, and can generally only be captured in specialized centers. (2) Examinations in lab environments show temporal and spatial restrictions. They cover a small time interval of the subject’s performance in a predefined environment (flat floor, no obstacles) as well as under a standardized inspection range. (3) All examinations are performed in a strange environment whilst being observed by a stranger (the doctor/investigator). This is an unnatural situation for the test subject. Mental pressure, an effort by the subject to perform well in the presence of an investigator, and little or no acclimatization period to the equipment and task are potential problems. Additionally, the subject might get used to the procedure after several task repetitions and then perform better during subsequent repetitions. As a result, it is highly likely that people walk differently in a controlled lab environment compared to a non-controlled real-world setting. This circumstance would hinder the extraction of a subject’s natural walking patterns and may falsify observations which would lead to the false diagnosis of particular diseases.

To address these issues, two main approaches have recently matured for capturing a subject’s walking patterns objectively and accurately in non-clinical settings: (1) Non-wearable systems, such as camera-based optical motion capture, or ground reaction force plates, and (2) wearable inertial measurement unit (IMU) sensor systems (Muro-de-la-Herran et al., 2014). The former requires considerable set-up time and equipment, and is generally restricted to lab environments or specialized centers. On the other hand, IMU sensors require less effort to set up for data collection outside the lab, and studies can be run without the need for direct observation of the test subject, thus enabling various real-world investigations to be undertaken (Yu et al., 2016; Figueiredo et al., 2018; Wang and Adamczyk, 2019).

With correct application of such objective approaches, it should therefore become possible to capture real-world characteristics of natural walking patterns that are able to inform clinical decision making through measurements in a non-controlled environment. As a result, such novel technologies could potentially support the early diagnosis of particular diseases. To this aim, several researchers have reported significant differences in gait parameters between controlled lab and non-controlled real-world settings (Weiss et al., 2011; Robles-García et al., 2015; Brodie et al., 2016; Del Din et al., 2016). However, these studies are difficult to compare since they all involve different parameters, test subjects and absolute error values in the assessment methods used. Therefore, our study aims to compare a broad spectrum of parameters using a validated estimation process on both young and elderly healthy subjects. We hypothesize that elderly subjects walk differently in a controlled lab environment compared to non-controlled real-world environments. Here, the young group serves as a control cohort to assess both whether the chosen IMU approach is sufficiently sensitive to detect differences in gait patterns between lab and real-world environments, but also whether any observed effect occurs across the entire population or rather simply in elderly subjects. Differences in walking patterns between the two environments would indicate potentially unnatural walking characteristics under lab conditions and can additionally emphasize differences between the age groups.

## Materials and Methods

### Subject Groups

Twenty young subjects (10 female, 10 male, 24.9 ± 2.7 years) and 20 elderly subjects (10 female, 10 male, 74.5 ± 8.6 years) were included in this study (Table 1). Subject inclusion criteria consisted of age (young: between 18 and 40 years, elderly: between 65 and 100 years) as well as no visible symptoms of any gait disorder, neurological disorder or cardiovascular disorder, which might affect normal standing or walking. Furthermore, female subjects during pregnancy or pre-menopausal state were excluded. All subjects provided their written, informed consent to participate in the study, which was approved by the Cantonal Ethics Committee Zurich (BASEC-No. 2018-00051) and Swissmedic (102597735).

**TABLE 1:** Subject characteristics (mean ± STD).

	**Young**	**Elderly**
Male	*n* = 10	*n* = 10
Female	*n* = 10	*n* = 10
Age (years)	24.9 ± 2.7	74.5 ± 8.6
Height (cm)	173.9 ± 9.5	171.4 ± 9.7
Weight (kg)	68.7 ± 13.4	70.7 ± 12.1

### Sensor Placement

**FIGURE 1:**
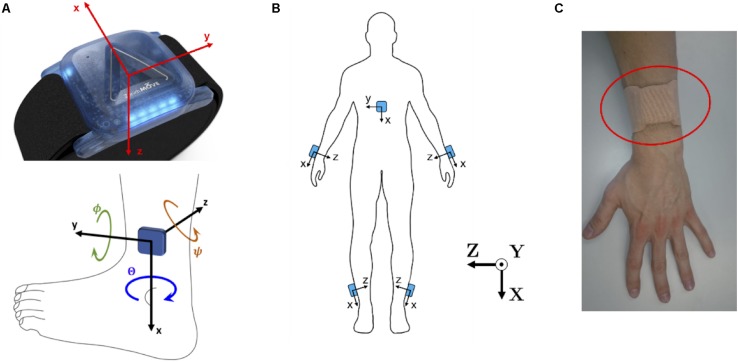
**(A)** ZurichMOVE sensor axis orientation for accelerometer and gyroscope as well as the three Euler Angles, together with positive turning direction, Θ around the *x*-axis, Φ around the *y*-axis and Ψ around the *z*-axis. **(B)** ZurichMOVE sensor attachment on both ankles, both wrists and the chest including the axis orientation. **(C)** Sensor attachment to the body using kinesiology tapes.

Five wearable ZurichMOVE^1^ IMU sensors (Schneider et al., 2018) were used for gait monitoring, with one attached to each ankle, and each wrist, as well as the chest, using kinesiology tape (Figure 1), to monitor axial acceleration *a*(*t*) and angular velocity ω(*t*) for each segment. The global *X*-axis was defined as the vertical axis, the global *Y*-axis as the anteroposterior axis and the global *Z*-axis as the lateral axis. The chosen attachment sites and taping method allowed the subjects to wear the sensors for several days without removal and with full freedom of movement.

### Tasks and Environment Under Investigation

While wearing the IMU sensors, all test subjects performed a calibration test for the step width estimation. They walked a distance of five meters on two parallel lines, with a specified spacing, repeating the procedure with a different line spacing (see section Gait Parameters). To assess gait in the controlled lab environment, subjects walked a marked distance of 10 m, four times (180° turnaround in-between) at their preferred walking speed. The test track in the lab was built on a flat floor with marked lines fixed to the floor as guidance. Subsequently, the sensors remained attached for 3 days to monitor the subjects’ walking patterns in their own real-world environment.

### Data Processing

The ZurichMOVE sensors use radio frequency time synchronization to prevent time-dependent drift between the individual sensors. The data were recorded at a frequency of over 1000 Hz and resampled at 50 Hz. Additionally, the axial acceleration values were low-pass filtered using a first-order Butterworth filter with a cut-off frequency of 5 Hz, while the angular velocity was filtered with a cut-off frequency of 12 Hz (settings adapted from Benoussaad et al., 2016). All data processing and calculations were performed using MATLAB (The MathWorks Inc., Natick, MA, United States).

### Automated Detection of Relevant Sequences

Out of the 3 days of recorded data, only sequences of walking, arm swinging and body turning were used for our analysis. To identify and extract these sequences efficiently, an automated detection process was developed, as described in the following sections.

#### Walking

**FIGURE 2:**
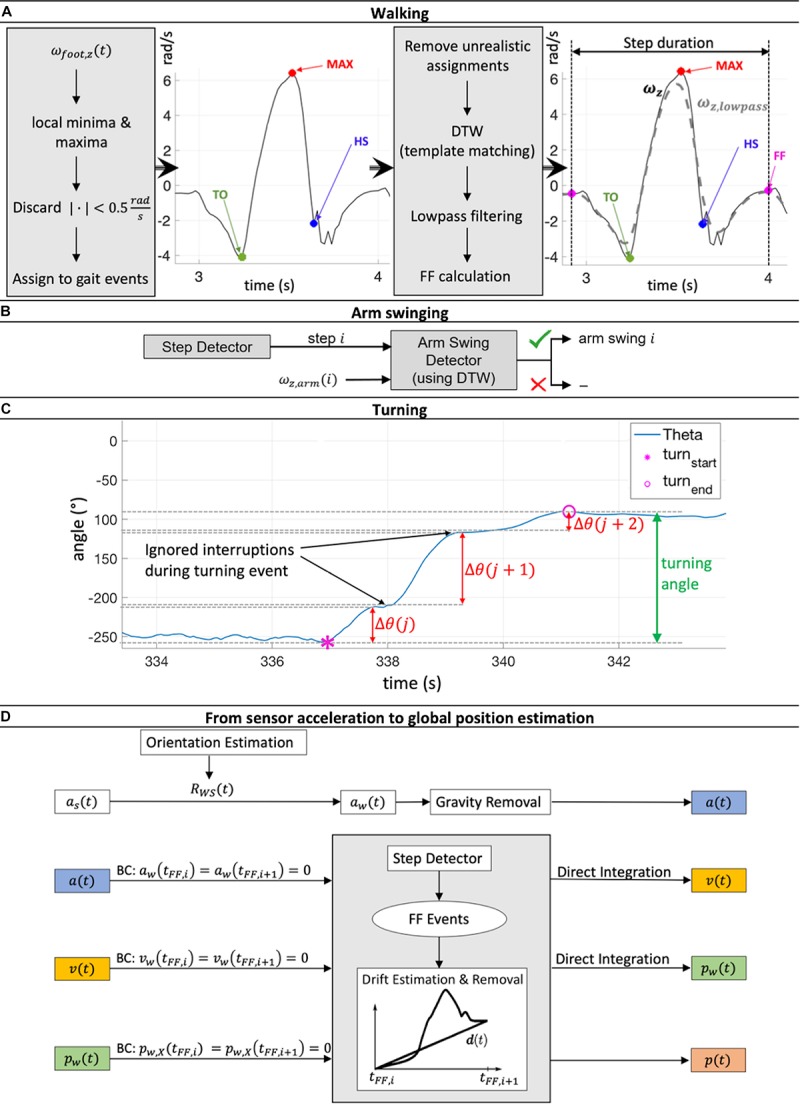
**(A)** Workflow of step detection based on a minima and maxima angular velocity search in the z-direction of the foot sensor followed by a dynamic time warping (DTW) based template matching procedure. **(B)** For every reported step, the presence or not of arm swinging was checked using DTW template matching. **(C)** The process of merging turning sequences Θ(j), Θ(j + 1) and Θ(j + 2) belonging to the same turning event to get the full turning angle is illustrated. **(D)** Estimation workflow of global acceleration ɑ(t), velocity v(t), and position p(t) during one gait cycle [between two foot flat (FF) events] via double integration of IMU acceleration data ɑ_*s*_(t). Rotation matrix *R*_*WS*_(*t*) then rotated the sensor position into global coordinates. Drift was linearly estimated and removed, including compensation for the effect of gravity. Zero acceleration and velocity at FF events and ground-level walking were assumed. This Integration process was performed for each gait cycle individually. BC, boundary conditions.

The monitoring of angular velocity in the z-direction of the foot sensor ω_*foot*,*z*_ has been identified as a viable method to detect gait events (Jasiewicz et al., 2006; Li et al., 2010), and was used in this study. As a result, gait cycles were considered to consist of a specific sequence of gait events (Figure 2A): (1) The gait cycle started at the foot flat (FF) event, when the leg was in a vertical position; (2) The start of swing phase started at the point when the toe lost contact with the ground (Toe-off, TO); (3) The time point when ω_*foot*,*z*_ was at its greatest was used to identify the maximum angular velocity (MAX) event; (4) Heel strike (HS) was defined as the point when the heel touched the ground, which terminated the swing phase and started the stance phase; (5) A subsequent FF event terminated the gait cycle.

The algorithm initially searched for local minima and maxima in order to detect the gait events mentioned above using the following restrictions: the time between two minima or maxima had to be at least 0.7 s, a peak had to be more than 0.5 rad/s larger in magnitude than the surrounding data, and the absolute magnitude of all minima and maxima had to be greater than 0.5 rad/s. Minima and maxima were then assigned to the different potential gait cycles. Sequences with unrealistic assignments (e.g., more than 10 s time difference between the assigned minima and maxima) were removed. These boundary conditions were set empirically based on previously recorded test data of normal walking.

At this stage, not all labeled movement patterns were considered to be “true steps” (steps that were complete and correctly met all criteria). To discard falsely classified steps, a template matching approach based on dynamic time warping (DTW) was applied, adapted from Li et al. (2012). Every potential step was compared with a predefined template step. The DTW procedure allows sequences of different magnitude and length to be checked for their similarity by calculating the DTW distance (see section “1. Dynamic time warping” in Supplementary Material and Müller, 2007). If this distance was below a threshold of 2.5, which was empirically set in our study based on our previously recorded test data of normal walking (shown to work reliably for straight walking, curvy walking as well as walking with slight gradients; The applied algorithm is available at http://doi.org/10.5905/ethz-1007-243), the step was reported as a true step and was included in the analysis (Figure 2A). Here, stairs ascent and descent led to DTW distance values larger than 2.5 and were therefore discarded. Evaluation of other special walking conditions was not performed since they did not occur often and were therefore considered to have no major influence on the averaged gait parameters over the 72 h time span of the investigation.

#### Arm Swinging

The presence of arm swinging was checked for every true step reported by the step detection algorithm. Similar to the step detection algorithm, a DTW based matching approach (against a predefined template arm swing) was applied to the angular velocity signal in the z-direction of the wrist sensor ω_*arm*,*z*_. If the DTW threshold was greater than 2.5, arm swinging was positively identified and entered into the analysis (Figure 2B).

#### Turning

Sequences of turning were identified by local integration of the angular velocity signal around the *x*-axis of the chest sensor ω_*chest*,*x*_. A turning sequence Δθ(*j*) was summed as long as no sign change of ω_*chest*,*x*_ was detected (the subject was turning in the same direction). If a sign change was observed (the subject was turning in the opposite direction), Δθ(*j*) was saved, and the integration process was reset such that a new turning sequence Δ(*j* + 1) was initiated. However, not every Δθ(*j*) directly represented one complete turning event. Due to interruptions while turning caused by e.g., step impacts, all turning sequences, Δθ, belonging to the same turning event had to be merged to obtain the full turning angle (Figure 2C), further details are shown in the section “2. Merging process of several turning sequences to full turning events” in Supplementary Material. One of our estimated gait parameters was the number of steps per 180° turn. To also compensate for errors during integration and merging processes, all Δθ larger than 160° were kept, and the number of steps during these turning events normalized to the number of steps taken to 180°.

### Gait Parameters

Our set of relevant gait parameters mainly consisted of standard spatial and temporal parameters commonly used in gait analysis such as stride length, gait velocity or cadence (Roberts et al., 2017). Since our gait analysis approach was motivated by the aim to identify people with signs of a walking disorder, we have included additional gait parameters that are indicative of a specific neurodegenerative disease such as NPH. For NPH patients, the following observations have been previously reported: increased foot outward rotation, increased number of steps needed for a 180° turnaround, increased cycle time deviation, and impaired arm swing compared to asymptomatic controls (Stolze et al., 2000, 2001; Relkin et al., 2005; Gallia et al., 2006; Shrinivasan et al., 2011). As a result, 15 parameters were used to capture the walking patterns of the subjects in this investigation (Table 2). To avoid the accumulation of errors due to the integration process, all parameters were calculated independently for every gait cycle,*i*, and the start position of integration was repetitively initialized to zero (Hamacher et al., 2014).

**TABLE 2:** Fifteen gait parameters were captured using the wearable ZurichMOVE IMU sensors.

**Symbol**	**Gait Parameter**	**Unit**	**Estimation Method**
*SL*	Stride length	m	Orientation estimation feet and double integration of *a_foot_*(*t*) during one gait cycle
*FC_max_*	Max foot clearance	cm	Orientation estimation feet and double integration of *a_foot_*(*t*) during one gait cycle
*V_Gait_*	Gait velocity	m/s	Orientation estimation feet and integration of *a_foot_*(*t*) during one gait cycle
Θ	Foot outward rotation	°	Use the ratio of the traveled foot displacement *d*_*lateral*_ and *d*_*anteroposterior*_
*SW*	Step width	cm	Check vertical tilting angle Φ_*foot*_(*t*) at FF events and extra calibration
*n_StepsTurning_*	Steps per 180° turn	−	Get turning sequences by local integration of ω_*chest,z*_(*t*) and detect steps in-between
*P_stance_*	Stance phase	% of gait cycle	Step detection algorithm based on ω_*foot,z*_(*t*)
*P_swing_*	Swing phase	% of gait cycle	Step detection algorithm based on ω_*foot*,*z*_(*t*)
*P_DL_*	Double limb support phase	% of gait cycle	Step detection algorithm based on ω_*foot*,*z*_(*t*)
*R_StanceToSwing_*	Stance to swing ratio	−	Step detection algorithm based on ω_*foot,z*_(*t*)
*n_cycle_*	Cadence	spm	Step detection algorithm based on ω_*foot,z*_(*t*)
*T_cycle_*	Cycle time	s	Step detection algorithm based on ω_*foot*,*z*_(*t*)
*dev*{*T_cycle_*}	Cycle time deviation	%	Step detection algorithm based on ω_*foot*,*z*_(*t*)
*A_swing,arm_*	Arm swing amplitude	rad/s	$\sqrt{\omega_{a\,r\,m,z}^{2}\,\left(t\right)+\omega_{a\,r\,m,y}^{2}\,\left(t\right)}$
*dist_arm_*	Traveled arm distance	m	Orientation estimation arm and double integration of *a_arm_*(*t*) during one gait cycle

*The estimation method of all parameters is briefly described in the last column.*

Temporal parameters were directly calculated in the sensor frame using the estimated gait events from the step detection. All spatial parameters were assessed using accelerations in the global (world) coordinate system as applied in similar successful approaches (Hamacher et al., 2014; Rampp et al., 2015; Benoussaad et al., 2016; Hannink et al., 2017). As a result, the sensor acceleration data *a*_*s*_(*t*) had to be expressed in global coordinates. This was achieved using a rotation matrix *R*_*WS*_(*t*) that identified how the sensor frame was oriented with respect to the global frame at every time instance,*t*. Orientation estimation was applied individually for each gait cycle and combined the acceleration and angular velocity measurements to obtain the rotation matrices, *R*_*WS*_(*t*) [similar to gyroscope integration (Hannink et al., 2017), described in section “3. Orientation estimation” in Supplementary Material]. Additionally, the effect of gravity was removed to obtain the global movement component of acceleration *a*(*t*):

(1) $$\begin{array}{c} a\,\left(t\right)=\left(R_{W\,S}\,\left(t\right)\cdot a_{s}\,\left(t\right)\right)+{\left[1,0,0\right]}^{T} \end{array}$$

To estimate the global position trajectory during the gait cycle *i*, *a*(*t*)was integrated twice (trapezoidal integration) between two FF events. In addition, the offset between foot and ankle was neglected during application of the following boundary conditions: the global acceleration *a*(*t*_*FF*_), velocity *v*(*t*_*FF*_) and vertical position *p*_*X*_(*t*_*FF*_) at ground contact during the FF event must be zero.

(2) $$a\,\left(t_{F\,F,i}\right)=a\,\left(t_{F\,F,i+1}\right)=v\,\left(t_{F\,F,i}\right)=v\,\left(t_{F\,F,i+1}\right)=0\;\&\;p_{X}\,\left(t_{F\,F,i}\right)=p_{X}\,\left(t_{F\,F,i+1}\right)=0$$

In order to ensure the constraint *a*(*t*_*FF*,*i*_) = *a*(*t*_*FF*,*i* + 1_) = 0, a drift estimation and removal (termed dedrifting) with a piecewise linear function as explained by Rampp et al. (2015) was applied to *a*(*t*) before the integration process. Due to inaccurate orientation estimation, sensor drift, and integration errors, the integrated signal *v*(*t*) does not necessarily satisfy the constraint *v*(*t*_*FF*,*i*_) = *v*(*t*_*FF*,*i* + 1_) = 0. Therefore, *v*(*t*) was dedrifted using the approach of Benoussaad et al. (2016):

(3) $$\begin{array}{c} v_{d\,e\,d\,r\,i\,f\,t\,e\,d}\,\left(t\right)=v\,\left(t\right)-\frac{v\,\left(t_{F\,F,i+1}\right)}{t_{F\,F,i+1}-t_{F\,F,i}}\cdot t \end{array}$$

where *t* is the time, *t*_*FF*,*i* + 1_−*t*_*FF*,*i*_ is the entire duration of the current gait cycle, and*v*(*t*_*FF*,*i* + 1_) is the calculated velocity at the end of the current gait cycle. After the second integration, *p*_*X*_(*t*) was dedrifted to fulfill the constraint *p*_*X*_(*t*_*FF*,*i*_) = *p*_*X*_(*t*_*FF*,*i* + 1_) = 0. The complete global position, *p*(*t*), estimation process is summarized in Figure 2D. All spatial feet parameters were estimated using *p*(*t*), except for step width, which required an additional calibration procedure due to the missing relative spatial relation between the IMU sensors. As an approximation, a linear reference line was defined to match sensor tilting angles at FF events Φ(*t*_*FF*_) to the width *d* between the heels:

(4) $$\begin{array}{c} d=w\cdot \Phi \,\left(t_{F\,F}\right)+c \end{array}$$

**FIGURE 3:**
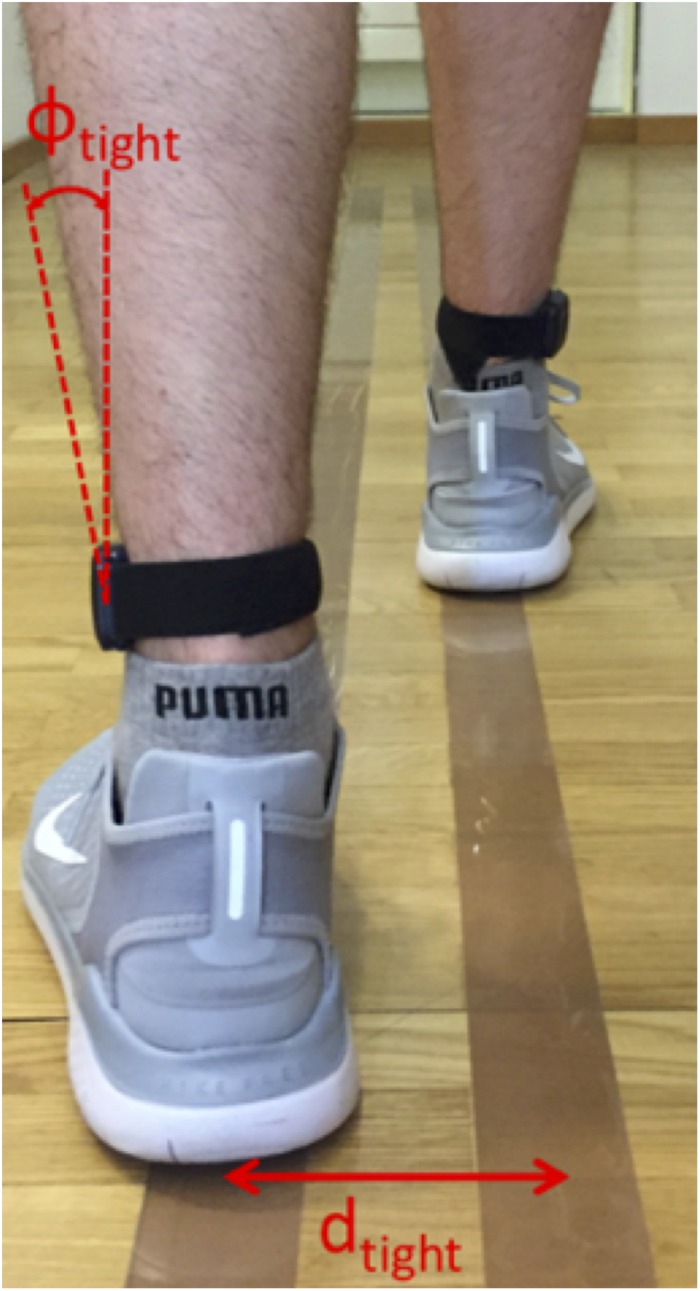
Principle of step width (SW) calibration procedure. The subject walked on two parallel lines, spaced by d_*tight*_ or d_*broad*_, for which the tilting angles Φ_*tight*_ and Φ_*broad*_ were evaluated. These four values were used to define a linear reference line for the SW estimation where the Φ values were matched to d values between the feet.

To define such a line, a calibration measurement was set up where the test subject walked on two lines with a known line spacing *d*, and the tilting angle Φ(*t*_*FF*_) of the sensors was evaluated for this walking sequence (Figure 3). The procedure was performed twice with different line spacings, *d*_*tight*_ (individual to subject), and *d*_*broad*_ (predefined upper limit of 35 cm). The two resulting calibration pairs *d*_*tight*_,Φ_tight_ and *d*_*broad*_,Φ_broad_ determined the parameters *w* and *c* of the reference line. To avoid unnatural gait patterns during these calibration trials, *d*_*tight*_ was not predefined but was rather found by visual inspection of the subject’s gait during a test walk of 5 m length. Furthermore, *d*_*broad*_ was visually corrected if the subject did not hold the default line spacing of 35 cm. The calibration was performed for every subject due to differences in anatomy and sensor alignment. After the calibration process, the step width (*SW*) was evaluated using the reference line:

(5) $$\begin{array}{c} S\,W=w\cdot \Phi \,\left(t_{F\,F}\right)+c \end{array}$$

Finally, the global coordinates *p*(*t*) of the wrist sensors were calculated by applying a workflow similar to that applied using the foot sensors (see Figure 2D), but without boundary conditions. The relative traveled arm distance was calculated in both the lateral (z) and anteroposterior (y) directions using the approach presented by Lewek et al. (2010). Details about the estimation of each parameter can be found in the section “4. Estimation of the 15 gait parameters” in Supplementary Material.

### Validation Experiment

We compared and verified our developed gait parameter estimation method against measurements using an opto-electronic motion capture system (Vicon motion analysis system, Oxford Metrics Group, United Kingdom) using 10 cameras to capture the movement trajectories of 61 markers attached to the body (see section “5. Validation measurement with Vicon” in Supplementary Material). For validation, three subjects with a total of 60 gait cycles were assessed. Here, each subject walked around a predefined test track in the shape of an eight around two cones (König et al., 2014) at their own preferred walking speed (normal walking conditions) while wearing the 61 markers as well as the five ZurichMOVE sensors. The two systems were time-synchronized and gait parameters were estimated independently for both systems.

The validation experiment revealed an accuracy of between 1% and 6% for most parameters, which was only slightly worse than attaching ZurichMOVE sensors directly to the foot (Mohammadi et al., 2017). Measured parameters with larger error values were checked using additional walking conditions (short, long, and broad walking). On completion of these verification activities, it became clear that all parameters and the corresponding error behavior could be described using a constant offset throughout the different conditions [*SW*: 39.3±5.7 cm (IMU), 31.6±4.7 cm (Vicon), 7.2±4.0 cm (abs diff) during broad walking; *T_DL_*: {0.29±0.08 s, 0.25±0.06 s} (IMU), {0.18±0.05 s, 0.14±0.03 s} (Vicon), {0.11±0.05 s, 0.10±0.03 s} (abs diff) during {short, long} step walking]. The reason for the constant offset of 1–2 steps in *n_StepsTurning_* between IMU estimation and visual inspection was differences in start and stop time definition of turning events: IMU estimation considered trunk rotation while visual inspection was focused on the feet only. As a result, all parameters developed were considered suitably verified to be used for relative comparisons between different test subjects and/or environments (Table 3).

**TABLE 3:** Results of the validation experiment during normal walking.

Parameter*	**Values**		**Absolute Error**	**Relative Error**
	**IMU**	**Vicon**	**Mean ± STD**	**Mean ± STD**
*SL* (m)	1.37 ± 0.14	1.33 ± 0.14	0.02 ± 0.03	1.6 ± 2.1%
*FC_max_*(cm)	11.7 ± 1.2	12.4 ± 1.7	−0.7 ± 1.4	−5.6 ± 11.2%
*V_Gait_* (m/s)	1.17 ± 0.22	1.19 ± 0.24	−0.01 ± 0.02	−0.8 ± 1.6%
Θ (°)	9.3 ± 2.6	9.5 ± 2.8	−0.2 ± 3.3	−1.9 ± 34.9%
*SW* (cm)	16.5 ± 4.7	7.6 ± 2.7	9.1 ± 4.4	118.4 ± 57.8%
n_StepsTurning_** (−)	7.2 ± 2.6	5.5 ± 3.0	1.7 ± 0.6	30.9 ± 10.9%
T_stance_*** (s)	0.69 ± 0.10	0.72 ± 0.09	−0.02 ± 0.03	−2.9 ± 4.5%
T_swing_*** (s)	0.46 ± 0.04	0.44 ± 0.03	0.02 ± 0.04	4.4 ± 8.5%
T_DL_*** (s)	0.24 ± 0.10	0.16 ± 0.04	0.09 ± 0.07	56.5 ± 43.3%
*n_cycle_* (spm)	105.3 ± 9.9	105.5 ± 8.6	−0.9 ± 4.5	−0.9 ± 4.3%
*T_cycle_* (s)	1.15 ± 0.12	1.16 ± 0.11	0.00 ± 0.03	−0.1 ± 2.9%
*dist_arm_* (m)	0.66 ± 0.19	0.67 ± 0.22	−0.01 ± 0.11	−0.8 ± 16.8%

The IMU sensors and Vicon markers were all attached to the body and the gait parameters were calculated independently with both systems. The absolute error ± standard deviation (STD) is presented as the difference between IMU and Vicon. Abbreviations are listed in Table 2. *R_*StanceToSwing*_, dev{T_*cycle*_} and A_*swing,arm*_ need no validation, directly calculated from validated parameters/sensor readings. **Visual inspection as reference. ***Period T is validated instead of P = T/T_*cycle*_ (% of gait cycle).

### Statistical Analyses

Each gait parameter was determined as the average of the left and right foot median values. For the evaluated gait parameters in the real-world environment, the following outlier removal was applied before the median calculation: (1) Walking phases were only considered if seven or more steps were performed in a row. (2) Times of special activities (e.g., sports) were reported by the test subjects in a protocol and removed from the analysis. (3) Values that deviated more than three times from the median value were discarded. Differences between the two environments and groups were evaluated as absolute difference (abs diff), calculated as Parameter_Real−world_−Parameter_Lab_, while mean relative difference (mean rel diff) was calculated as the mean of the relative differences (Parameter_Real−world_-Parameter_Lab_)/(*Parameter*_Real−world_). All analyses were performed using MATLAB (The MathWorks Inc., Natick, MA, United States). The resulting median values of each subject were then compared between the different environments, and tested for significance using the Wilcoxon signed-rank test, while differences between the two test groups (young and elderly) were examined using the Mann–Whitney *U*-Test. To ensure symmetrical data distribution (assumption of Wilcoxon signed-rank test), parameters with a skewed distribution were log transformed before *p*-value calculation. Since our hypotheses include several parameters, resulting *p*-values were corrected for false discovery by applying the Benjamini-Hochberg correction. All statistical tests were performed in R (R Core Team, 2017, Vienna, Austria), with *p*-values smaller than 0.05 considered to be statistically significant.

## Results

### Non-controlled Real-World Versus Controlled Lab Environment

**FIGURE 4:**
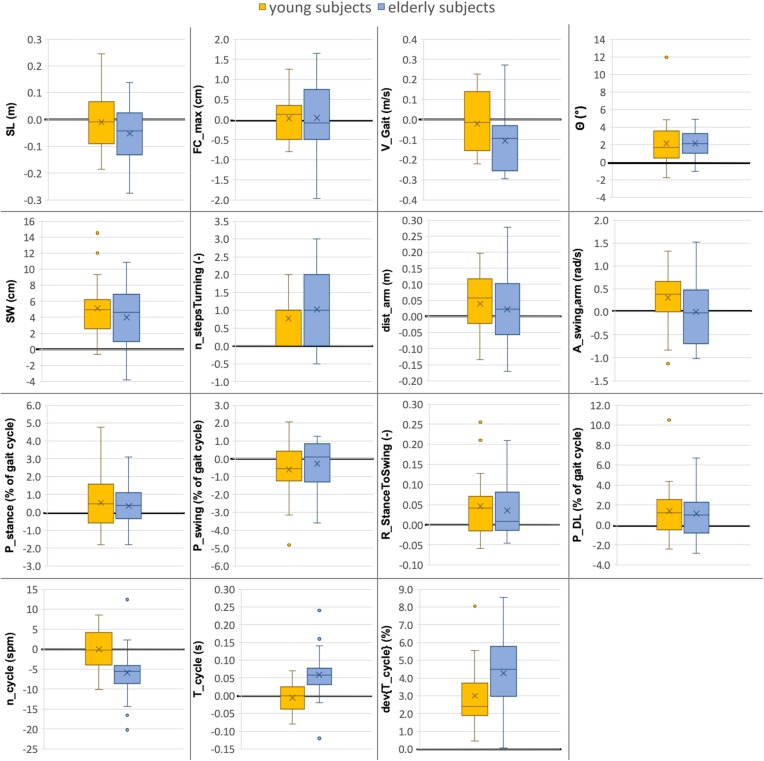
Comparison between gait parameters collected in lab (10-m walking test) versus real-world (72 h investigation) environments for young and elderly subjects (*n* = 20 each). The boxplots indicate the absolute differences between the two environments (Parameter_*Real–world*_ - Parameter_*Lab*_) for both groups. The median value is illustrated as a line, the mean value as a cross and outliers as dots. The line indicating zero difference between the two settings is depicted in bold. Abbreviations are listed in Table 2.

Based on the results (Table 4 and Figure 4), the parameters were divided into three clusters: (A) Significant differences between the two environments for both groups; (B) Significant differences between the two environments for the elderly subjects only; (C) Remaining differences.

**TABLE 4:** Estimated gait parameters of young and elderly test subjects (*n* = 20 each) in real-world and lab environment (mean ± standard deviation) clustered in A, B and C.

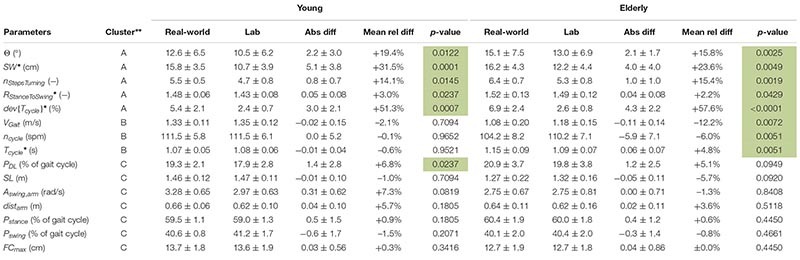

Absolute (abs diff) and mean relative differences (mean rel diff) between the non-controlled real-world and controlled lab environments are presented. *P*-values are calculated based on the abs diff data, resulting values smaller than 0.05 are considered statistically significant (marked in green). Respective abbreviations are listed in Table 2. *Skew distributed data, transformed logarithmically for *p*-value calculation (Wilcoxon signed-rank test requires symmetric data distribution). **(A) Significant differences between the two environments for both groups; **(B)** Significant differences between the two environments for elderly group only; **(C)** Remaining differences.

#### Cluster A: Significant Differences Between the Two Environments for Both Groups

In the real-world settings, both groups showed a significantly increased foot outward rotation [young: 19% (*p* = 0.0122); elderly: 16% (*p* = 0.0025)], a step width increase [young: 32% (*p* = 0.0001); elderly: 24% (*p* = 0.0049)], an increased number of steps per 180° turn [young: 14% (*p* = 0.0145); elderly: 15% (*p* = 0.0019)] for elderly subjects, an increased cycle time deviation [young: 51% (*p* = 0.0007); elderly: 58% (*p* < 0.0001)] and an increased stance to swing ratio [young: 3% (*p* = 0.0237); elderly: 2% (*p* = 0.0429)].

#### Cluster B: Significant Differences Between the Two Environments for Elderly Group Only

Several parameters showed a larger difference between the two environments for elderly subjects than for the young. For the elderly, we observed a 12% decrease in gait velocity (*p* = 0.0072), a 5% increase in cycle time (*p* = 0.0051) and a 6% decrease in cadence (*p* = 0.0051) in the real-world compared with the lab environment. For the young subjects, these differences were minor and non-significant with values of −2, −1, and ±0%, respectively.

#### Cluster C: Remaining Differences

The double limb support phase showed significant differences between the two environments for young subjects [7% (*p* = 0.0237)] in real-world settings while the increase for elderly subjects was not statistically significant (5%). Although not significant in both groups, similarity to the parameters of Cluster A is present. Furthermore, non-significant differences between the two environments were an increased traveled arm distance (young: 6%; elderly: 4%) as well as a decreased stride length (young: −1%; elderly: −6%) in real-world settings. The *p*-values of the stride length are much smaller in elderly subjects compared with the young which indicates a potential link to Cluster B. The arm swing amplitude was the only parameter that was considerably increased in young subjects (7%) in the real-world environment compared to a decrease in elderly subjects (−1%). Foot clearance, stance phase, and swing phase did not show relevant differences between the two environments for either group (smaller than 1.5%).

### Young Versus Elderly Test Subjects

Overall, the non-controlled real-world environment *enlarged* the inter-group differences. In both environments, young test subjects took significantly longer strides (*p* = 0.0047 real-world, *p* = 0.0063 lab) and walked faster (*p* = 0.0001 in real-world, *p* = 0.0063 in lab environment) than elderly subjects. In real-world settings, young subjects also took significantly less steps per 180° turn (*p* = 0.0024), walked with higher cadence (*p* = 0.0120), and showed an increased cycle time (*p* = 0.0110) compared with elderly subjects. The remaining parameters did not show significant differences between the two test groups in either environment.

## Discussion

The current study compared the walking patterns of young and elderly subjects in a controlled lab environment against those captured in a non-controlled real-world environment over 72 h. Significant differences were present between the two environments for both groups, including increased foot outward rotation, step width, number of steps per 180° turn, stance to swing ratio, and cycle time deviation in real-world settings. Although only significant in young subjects, both groups also exhibited an increased double limb support phase in real-world environments. Furthermore, we observed significant differences between the two environments only in the elderly subjects, including decreased gait velocity, decreased cadence, and increased cycle time in real-world settings. In general, the young subjects showed only minor differences in their walking patterns between the two environments. Additionally, the gait parameters were compared between the two test groups for both environments, where the non-controlled real-world environment enlarged the inter-group differences.

The significant differences in several gait parameters between the two environments confirm our hypothesis that people walk differently in a controlled lab environment. The differences in walking patterns between the two environments were minor for young test subjects compared to the elderly, suggesting that elderly subjects tend to be more influenced in their walking patterns by their environment (Del Din et al., 2016) as well as the possible surveillance of an independent audience (Robles-García et al., 2015; Brodie et al., 2016). This observation may indicate that elderly subjects in particular tend to perform better in a controlled lab environment because they try not to stand out negatively during a test or survey (Del Din et al., 2016). The observed differences in Cluster B match several results of other studies. Brodie et al. (2016) observed a trend toward lower cadence in real-world environments for elderly people, while Del Din et al. (2016) reported a decreased stride length, decreased gait velocity, and increased cycle time for elderly subjects and subjects suffering from Parkinson’s disease in real-world settings. Furthermore, an increased cycle time deviation in real-world environments has been reported for elderly people (Brodie et al., 2016; Del Din et al., 2016) as well as an increased variability in cadence (Weiss et al., 2011). For Cluster A, no reports about similar behavior between the two environments were found in the literature, possibly reflective the bias toward reporting only positive results.

We see two main reasons for the observed differences: a mental and a physical influence of the environment. The mental status of the subject is known to influence walking behavior (Prohaska et al., 2009), while the physical influence is given by the various path characteristics, such as surface type, length and type of walking distance (e.g., straight/curved). For Cluster A, it is plausible that the physical influence of the environment plays a dominant role, since similar differences in gait parameters were present in both test groups. Perfect straight walking is possible within a controlled lab environment, but such conditions can rarely be assumed in ecologically valid real-world settings. It is likely that the differences observed in Cluster B were driven by the mental influence of the environment, as differences between the two environments were observed only in the elderly population. It is clear that the differences observed in Cluster B necessitate an improved understanding of natural walking patterns under ecologically valid conditions, including the role they play in clinical decision making.

Several parameters showed larger differences between young and elderly subjects for the non-controlled real-world environment. If this effect is also present in people with indications of a walking disorder, the enlarged separation between groups would be beneficial for diagnostic processes. Del Din et al. (2016) have already reported enlarged inter-group differences between elderly healthy controls and subjects suffering from Parkinson’s disease in real-world settings. However, further studies including people with various indications of walking disorder are needed to investigate this topic.

To ensure exact sensor positioning throughout the entire recordings, the subjects did not remove the sensors at any time. To allow wearing the sensors without removal and guarantee full freedom of movement, the sensors were attached to the ankles instead of the feet. Although the accuracy of gait parameter estimation of ankle mounted sensors is lower than of foot mounted sensors (mainly due to the lack of a stationary instant during the gait cycle) several researchers have investigated ankle mounted gait analysis and confirmed its validity for gait parameter estimation. Jasiewicz et al. (2006) estimated HS and TO events similar to our approach and reported high levels of accuracy for normal gait, but showed inaccuracies for abnormal gait e.g., using walking aids. Li et al. (2010) used an ankle mounted IMU sensor to estimate the gait velocity, Benoussaad et al. (2016) estimated the foot clearance and Sijobert et al. (2015) estimated stride length, all of them with comparable accuracy to our approach. Our findings regarding the comparison of walking patterns of young and elderly subjects agree with several reports from literature. For young subjects, an increased stride length and gait velocity (Whittle, 1991; Öberg et al., 1993; Prince et al., 1997; Menz et al., 2004; Janeh et al., 2018), an increased heel clearance in young subjects (Mariani et al., 2010), less time for double limb support phase (Aminian et al., 2002; Janeh et al., 2018), and more steps taken for turning in elderly subjects (Thigpen et al., 2000; Akram et al., 2010) have all been reported and are in agreement with the results of our study. Consequently, we are confident not only that our metrics determined in ecologically valid settings are reliable, but also that the comparison against lab-based settings has revealed valid differences between the settings. As a result, clinicians should be aware of the reported changes in movement patterns, especially in cases where gait metrics play a role in the diagnosis of a patient’s functional status e.g., fall risk, or when therapies require tuning to optimize muscle function and coordination e.g., deep brain stimulation.

One limitation during the assessment of the subjects’ walking patterns was the range of considered gait parameters. The current study mainly focused on gait parameters that capture the gait rhythm and pace of a subject. To extend the captured walking pattern range, asymmetry or variability could be included or investigated in parameters other than only cycle time (König et al., 2014; Del Din et al., 2016; McArdle et al., 2019). Additionally, variability in real-world environments could be captured in more detail by individual evaluation and averaging of every walking sequence, as proposed by Del Din et al. (2016). On the estimation side, the following deficiencies were present in our study: (1) The parameters with relative error values larger than 6% (*SW*,*T_DL_* and *n_StepsTurning_*) have to be used with caution. (2) Our approach needs a calibration procedure for the step width estimation for every subject, which may be a potential source of error. (3) Although our predefined line spacing during step width measurement calibration only acted as guidance, some subjects still focused too much on them, which may have falsified their natural walking pattern. This might be a potential reason for the large error in the step width estimation. (4) The foot outward rotation estimation was prone to sensor misalignment. Therefore, the sensors need to be attached to the body precisely, with their position maintained throughout the measurement period. (5) To avoid bias of the findings, we did not observe subjects in the real-world environment as we hypothesized that such observations may influence the gait patterns. However, potential differences in daily activities between the subjects remain unknown and the ability to extrapolate results beyond the examined metrics is therefore limited. In order to minimize this influence, we recorded activity over an extended 3 days period, but also ensured that the subjects filled in an activity protocol so that we were able to ignore all non-continuous walking sequences (less than seven steps in a row). (6) Our step detection approach did not differentiate between normal walking and slope ascent/descent walking. While such gait patterns could influence the event recognition, we expected most steps to be performed under conditions with negligible slope effects, with any exclusion of such steps serving to present conservative results. Finally, besides the considered influence of the environment on the walking pattern, further factors may exist, such as the influence of the attached sensors on the movement or a potential feeling of being surveyed by the sensors. Despite these limitations, we could show that people walk differently in a controlled lab environment, which should be considered during future examinations on gait characteristics regarding natural walking pattern extraction.

## Conclusion

We conclude that especially elderly subjects walked differently in controlled lab settings compared to their real-world environments. Elderly subjects tend to walk faster and take less time per step (increased cadence and decreased cycle time) in the controlled lab environment, whereas for young people, these differences were minor. The findings indicate the need to better understand natural walking patterns under ecologically valid conditions before clinically relevant conclusions can be drawn on a subject’s functional status. Moreover, the greater inter-group differences in real-world environments seem promising regarding the identification of subjects with indications of a walking disorder.

## Data Availability Statement

The developed step detection algorithm is available at http://doi.org/10.5905/ethz-1007-243 including a test walking sequence of ankle mounted angular velocity in the lateral direction. The datasets of the study are not publicly available because test subjects was guaranteed that their data will be anonymously used for this study only and not passed to extern instances. Requests to access the datasets should be directed to MS, marischm@ethz.ch.

## Ethics Statement

The studies involving human participants were reviewed and approved by the Cantonal Ethics Committee Zurich (BASEC-No. 2018-00051) and Swissmedic (102597735). The participants provided their written informed consent to participate in this study.

## Author Contributions

DR and NT contributed to the concept and design of the parameter estimation methods. DR, CG, and NS contributed to the concept and design of the Vicon validation. DR, CG, MS, and LS contributed to the concept and study design. DR and CG contributed to the data analysis and interpretation. DR and MS contributed to the drafting of the manuscript. DR, CG, NT, NS, MM, WT, LS, and MS contributed to the critical revision of the article. DR, CG, NT, NS, MM, WT, LS, and MS contributed to the approval of the manuscript.

## Conflict of Interest

The authors declare that the research was conducted in the absence of any commercial or financial relationships that could be construed as a potential conflict of interest.

## Footnotes

1 zurichmove.com

## References

[B1] Akram S. B. Frank J. S. Chenouri S. (2010). Turning behavior in healthy older adults: is there a preference for step versus spin turns? *Gait Posture* 31 23–26. 10.1016/j.gaitpost.2009.08.238 19765996

[B2] Allan L. M. Ballard C. G. Burn D. J. Kenny R. A. (2005). Prevalence and severity of gait disorders in Alzheimer’s and Non-Alzheimer’s dementias. *J. Am. Geriatr. Soc.* 53 1681–1687. 10.1111/j.1532-5415.2005.53552.x 16181166

[B3] Aminian K. Leyvraz P.-F. Robert P. Najafi B. Büla C. (2002). Spatio-temporal parameters of gait measured by an ambulatory system using miniature gyroscopes. *J. Biomech.* 35 689–699. 10.1016/s0021-9290(02)00008-8 11955509

[B4] Benoussaad M. Sijobert B. Mombaur K. Azevedo Coste C. (2016). Robust foot clearance estimation based on the integration of foot-mounted IMU acceleration data. *Sensors* 16:12. 10.3390/s16010012 26703622 PMC4732045

[B5] Bradley W. G. Whittemore A. R. Watanabe A. S. Davis S. J. Teresi L. M. Homyak M. (1991). Association of deep white matter infarction with chronic communicating hydrocephalus: implications regarding the possible origin of normal-pressure hydrocephalus. *Am. J. Neuroradiol.* 12 31–39. 1899515 PMC8367539

[B6] Brodie M. A. D. Coppens M. J. M. Lord S. R. Lovell N. H. Gschwind Y. J. Redmond S. J. (2016). Wearable pendant device monitoring using new wavelet-based methods shows daily life and laboratory gaits are different. *Med. Biol. Eng. Comput.* 54 663–674. 10.1007/s11517-015-1357-9 26245255

[B7] Del Din S. Godfrey A. Galna B. Lord S. Rochester L. (2016). Free-living gait characteristics in ageing and Parkinson’s disease: impact of environment and ambulatory bout length. *J. Neuroeng. Rehabil.* 13 1–12. 10.1186/s12984-016-0154-5 27175731 PMC4866360

[B8] Figueiredo J. Félix P. Costa L. Moreno J. C. Santos C. P. (2018). Gait event detection in controlled and real-life situations: repeated measures from healthy subjects. *IEEE Trans. Neural Syst. Rehabil. Eng.* 26 1945–1956. 10.1109/TNSRE.2018.2868094 30334739

[B9] Gallia G. L. Rigamonti D. Williams M. A. (2006). The diagnosis and treatment of idiopathic normal pressure hydrocephalus. *Nat. Clin. Pract. Neurol.* 2 375–381. 10.1038/ncpneuro0237 16932588

[B10] Hamacher D. Hamacher D. Taylor W. R. Singh N. B. Schega L. (2014). Towards clinical application: repetitive sensor position re-calibration for improved reliability of gait parameters. *Gait Posture* 39 1146–1148. 10.1016/j.gaitpost.2014.01.020 24602974

[B11] Hannink J. Ollenschläger M. Kluge F. Roth N. Klucken J. Eskofier B. M. (2017). Benchmarking foot trajectory estimation methods for mobile gait analysis. *Sensors* 17:1940. 10.3390/s17091940 28832511 PMC5621093

[B12] Hebb A. O. Cusimano M. D. (2001). Idiopathic normal pressure hydrocephalus: a systematic review of diagnosis and outcome. *Neurosurgery* 49 1166–1186. 10.1227/00006123-200111000-00028 11846911

[B13] Janeh O. Bruder G. Steinicke F. Gulberti A. Poetter-Nerger M. (2018). Analyses of gait parameters of younger and older adults during (Non-) isometric virtual walking. *IEEE Trans. Vis. Comput. Graph.* 24 2663–2674. 10.1109/TVCG.2017.2771520 29990158

[B14] Jaraj D. Rabiei K. Marlow T. Jensen C. Skoog I. Wikkelsø C. (2014). Prevalence of idiopathic normal-pressure hydrocephalus. *Neurology* 82 1449–1454. 10.1212/WNL.0000000000000342 24682964 PMC4001197

[B15] Jasiewicz J. M. Allum J. H. J. Middleton J. W. Barriskill A. Condie P. Purcell B. (2006). Gait event detection using linear accelerometers or angular velocity transducers in able-bodied and spinal-cord injured individuals. *Gait Posture* 24 502–509. 10.1016/j.gaitpost.2005.12.017 16500102

[B16] König N. Singh N. B. Baumann C. R. Taylor W. R. (2016a). Can gait signatures provide quantitative measures for aiding clinical decision-making? A systematic meta-analysis of gait variability behavior in patients with Parkinson’s disease. *Front. Hum. Neurosci.* 10:319. 10.3389/fnhum.2016.00319 27445759 PMC4927578

[B17] König N. Singh N. B. von Beckerath J. Janke L. Taylor W. R. (2014). Is gait variability reliable? An assessment of spatio-temporal parameters of gait variability during continuous overground walking. *Gait Posture* 39 615–617. 10.1016/j.gaitpost.2013.06.014 23838361

[B18] König N. Taylor W. R. Baumann C. R. Wenderoth N. Singh N. B. (2016b). Revealing the quality of movement: a meta-analysis review to quantify the thresholds to pathological variability during standing and walking. *Neurosci. Biobehav. Rev.* 68 111–119. 10.1016/j.neubiorev.2016.03.035 27189783

[B19] Lewek M. D. Poole R. Johnson J. Halawa O. Huang X. (2010). Arm swing magnitude and asymmetry during gait in the early stages of Parkinson’s disease. *Gait Posture* 31 256–260. 10.1016/j.gaitpost.2009.10.013 19945285 PMC2818433

[B20] Li F. Zhao C. Ding G. Gong J. Liu C. Zhao F. (2012). “A reliable and accurate indoor localization method using phone inertial sensors,” in *Proceedings of the 2012 ACM Conference on Ubiquitous Computing*, Montbeliard-Belfort.

[B21] Li Q. Young M. Naing V. Donelan J. M. (2010). Walking speed estimation using a shank-mounted inertial measurement unit. *J. Biomech.* 43 1640–1643. 10.1016/j.jbiomech.2010.01.031 20185136

[B22] Mariani B. Hoskovec C. Rochat S. Büla C. Penders J. Aminian K. (2010). 3D gait assessment in young and elderly subjects using foot-worn inertial sensors. *J. Biomech.* 43 2999–3006. 10.1016/j.jbiomech.2010.07.003 20656291

[B23] McArdle R. Galna B. Donaghy P. Thomas A. Rochester L. (2019). Do Alzheimer’s and Lewy body disease have discrete pathological signatures of gait? *Alzheimers Dement.* 15 1367–1377. 10.1016/j.jalz.2019.06.4953 31548122

[B24] Menz H. B. Latt M. D. Tiedemann A. Kwan M. M. S. Lord S. R. (2004). Reliability of the GAITRite^®^ walkway system for the quantification of temporo-spatial parameters of gait in young and older people. *Gait Posture* 20 20–25. 10.1016/S0966-6362(03)00068-7 15196515

[B25] Mohammadi M. Singh N. B. Hitz M. Orter S. Taylor W. R. Frigo C. (2017). “Achieving ecological validity in mobility assessment: validating a wearable sensor technology for comprehensive gait assessment,” in *Proceedings of the 2017 IEEE 3rd International Forum on Research and Technologies for Society and Industry (RTSI)*, Modena.

[B26] Müller M. (2007). *Information Retrieval for Music and Motion.* Berlin: Springer.

[B27] Muro-de-la-Herran A. García-Zapirain B. Méndez-Zorrilla A. (2014). Gait analysis methods: an overview of wearable and non-wearable systems, highlighting clinical applications. *Sensors* 14 3362–3394. 10.3390/s140203362 24556672 PMC3958266

[B28] Öberg T. Karsznia A. Öberg K. (1993). Basic gait parameters: reference data for normal subjects, 10-79 years of age. *J. Rehabil. Res. Dev.* 30 210—-210. 10.21595/jve.2017.18459 8035350

[B29] Prince F. Hkbert R. Winter A. (1997). Review article gait in the elderly corriveau. *Gait Posture* 5 128–135. 10.1016/s0966-6362(97)01118-1

[B30] Prohaska T. R. Eisenstein A. R. Satariano W. A. Hunter R. Bayles C. M. Kurtovich E. (2009). Walking and the preservation of cognitive function in older populations. *Gerontologist* 49 S86–S93. 10.1093/geront/gnp079 19525221

[B31] R Core Team (2017). *R: A Language and Environment for Statistical Computing.* (Vienna, Austria: R Foundation for Statistical Computing).

[B32] Rampp A. Barth J. Schülein S. Gaßmann K. G. Klucken J. Eskofier B. M. (2015). Inertial sensor-based stride parameter calculation from gait sequences in geriatric patients. *IEEE Trans. Biomed. Eng.* 62 1089–1097. 10.1109/TBME.2014.2368211 25389237

[B33] Ravi D. K. Gwerder M. Ignasiak N. K. Baumann C. R. Uhl M. van Dieën J. H. (2019). Revealing the optimal thresholds for movement performance: a systematic review and meta-analysis to benchmark pathological walking behaviour. *Neurosci. Biobehav. Rev.* 108 24–33. 10.1016/J.NEUBIOREV.2019.10.008 31639377

[B34] Relkin N. Marmarou A. Klinge P. Bergsneider M. Black P. M. L. (2005). INPH guidelines, part II: diagnosing idio-pathic normal-pressure hydrocephalus. *Neurosurgery* 57 4–16. 10.1227/01.NEU.0000168185.29659.C5 16160425

[B35] Roberts M. Mongeon D. Prince F. (2017). Biomechanical parameters for gait analysis: a systematic review of healthy human gait. *Phys. Ther. Rehabil.* 4:6 10.7243/2055-2386-4-6

[B36] Robles-García V. Corral-Bergantiños Y. Espinosa N. Jácome M. A. García-Sancho C. Cudeiro J. (2015). Spatiotemporal gait patterns during overt and covert evaluation in patients with Parkinson’s disease and healthy subjects: is there a Hawthorne effect? *J. Appl. Biomech.* 31 189–194. 10.1123/jab.2013-0319 25536440

[B37] Schneider S. Popp W. L. Brogioli M. Albisser U. Demkó L. Debecker I. (2018). Reliability of wearable-sensor-derived measures of physical activity in wheelchair-dependent spinal cord injured patients. *Front. Neurol.* 9:1039. 10.3389/fneur.2018.01039 30619026 PMC6295582

[B38] Shrinivasan A. Brandt-Pearce M. Barth A. Lach J. (2011). “Analysis of gait in patients with normal pressure hydrocephalus,” in *Proceedings of the First ACM Workshop on Mobile Systems, Applications, and Services for Healthcare*, Seattle.

[B39] Sijobert B. Benoussaad M. Denys J. Pissard-Gibollet R. Geny C. Coste C. A. (2015). Implementation and validation of a stride length estimation algorithm, using a single basic inertial sensor on healthy subjects and patients suffering from Parkinson’s disease. *Health* 7 704–714. 10.4236/health.2015.76084 15759579

[B40] Stolze H. Kuhtz-Buschbeck J. P. Drücke H. Jöhnk K. Diercks C. Palmié S. (2000). Gait analysis in idiopathic normal pressure hydrocephalus - which parameters respond to the CSF tap test? *Clin. Neurophysiol.* 111 1678–1686. 10.1016/S1388-2457(00)00362-X 10964082

[B41] Stolze H. Kuhtz-Buschbeck J. P. Drücke H. Jöhnk K. Illert M. Deuschl G. (2001). Comparative analysis of the gait disorder of normal pressure hydrocephalus and Parkinson’s disease. *J. Neurol. Neurosurg. Psychiatry* 70 289–297. 10.1136/jnnp.70.3.289 11181848 PMC1737236

[B42] Thigpen M. T. Light K. E. Creel G. L. Flynn S. M. (2000). Turning difficulty characteristics of adults aged 65 years or older. *Phys. Ther.* 80 1174–1187. 10.1093/ptj/80.12.1174 11087304

[B43] Wang W. Adamczyk P. G. (2019). Analyzing gait in the real world using wearable movement sensors and frequently repeated movement paths. *Sensors* 19:1925. 10.3390/s19081925 31022889 PMC6515355

[B44] Weiss A. Sharifi S. Plotnik M. Van Vugt J. P. P. Giladi N. Hausdorff J. M. (2011). Toward automated, at-home assessment of mobility among patients with Parkinson disease, using a body-worn accelerometer. *Neurorehabil. Neural Repair* 25 810–818. 10.1177/1545968311424869 21989633

[B45] Whittle M. W. (1991). *Gait Analysis: An Introduction.* Oxford: Butterworth-Heinemann.

[B46] Yu G. Jang Y. J. Kim J. Kim J. H. Kim H. Y. Kim K. (2016). Potential of IMU sensors in performance analysis of professional alpine skiers. *Sensors* 16 1–21. 10.3390/s16040463 27043579 PMC4850977

