# Supplementary material for: Wearable Inertial Measurement Units for Assessing Gait in Real-World Environments
Source: Front Physiol. 2020 Feb 20;11:90. doi: 10.3389/fphys.2020.00090 (PMC7044412; doi:10.3389/fphys.2020.00090)
Supplement: Supplementary file 1 [file Data_Sheet_1.pdf]

Supplementary Material

1 Dynamic time warping (DTW)

The DTW distance for two normalized time sequences X of length l_x and Y of length l_y is calculated as follows:

1. Define a cost matrix of size $n \times m$, where $n = \min\{l_x, 2w+1\}$, $m = \min\{l_y, 2w+1\}$ and w as window parameter for maximal window size restriction.
2. Calculate the Euclidean distances of all points i and j in the sequences X and Y and add the minimum of the previously calculated distances D (fill up the cost matrix):

$$D(i,j) = |\mathrm{X}(i) - \mathrm{Y}(i)| + \min\{D(i-1,j-1), D(i-1,j), D(i,j-1)\} \tag{1}$$

3. Calculate the DTW distance of X and Y by summation of entries $D(i,j)$ of the cost matrix along the optimal path (minimal costs):

$$\mathrm{DTW}(\mathrm{X},\mathrm{Y}) = \sum_{i,j\,\in \mathrm{optimal\ path}} D(i,j) + \min\{D(i-1,j-1), D(i-1,j), D(i,j-1)\} \tag{2}$$

   starting from $i = n, j = m$

In our study, we used w=25 samples (= 0.5 s at 50 Hz sampling frequency). For more details about DTW refer to Müller (2007).

2 Merging process of several turning sequences to full turning events

We developed a threshold-based algorithm to merge all estimated turning sequences $\Delta\theta$ belonging to the same turning event:

1. Discard all $\Delta\theta$ with $|\Delta\theta| < \mathrm{T1}$ with $\mathrm{T1} = 1°$
2. Combine turning sequence $\Delta\theta(j)$ and $\Delta\theta(j+1)$ if $\mathrm{sign}(\Delta\theta(j)) = \mathrm{sign}(\Delta\theta(j+1))$
3. Remove short in between interruptions $\Delta\theta(j)$ if all temporal (T2) and spatial thresholds (T3A, T3B) are fulfilled:
   - $t_{start,j+1} - t_{end,j-1} < \mathrm{T2}$ with $\mathrm{T2} = 1$ s
   - $|\Delta\theta(j-1)| > \mathrm{T3A}$ and $|\Delta\theta(j+1)| > \mathrm{T3A}$ with $\mathrm{T3A} = 20°$
   - $|\Delta\theta(j)| < \mathrm{T3B}$ with $\mathrm{T3B} = 5°$

   ending up in k turning sequences $\Delta\theta$

4. Keep turning events larger than 180° by some margin T4 = 20°: $|\Delta\theta(j)| + T4 > 180°$

3 Orientation estimation

Orientation estimation is used to calculate the sensor frame orientation with respect to the global world frame, expressed as rotation matrix R_{WS}. We follow the approach of Hannink et al. (2017). Due to simplicity, we use quaternions and transform the result to a rotation matrix in the end.

A quaternion represents a rotation of an angle θ around an axis $\boldsymbol{n}$:

$$\boldsymbol{q} = [q_0, \mathrm{q}_1, \mathrm{q}_2, q_3]^T = [\cos(\theta/2), \sin(\theta/2)\boldsymbol{n}]^T \tag{3}$$

A combination of several rotations is done by the (non-commutative) quaternion multiplication (denoted by ⊗). A quaternion rotation **r** followed by a quaternion rotation $\boldsymbol{q}$ is defined as

$$\boldsymbol{q} \otimes \mathbf{r} = \begin{bmatrix} q_0 \\ q_1 \\ q_2 \\ q_3 \end{bmatrix} \otimes \begin{bmatrix} r_0 \\ r_1 \\ r_2 \\ r_3 \end{bmatrix} = \begin{bmatrix} r_0 q_0 - r_1 q_1 - r_2 q_2 - r_3 q_3 \\ r_0 q_1 + r_1 q_0 - r_2 q_3 + r_3 q_2 \\ r_0 q_2 + r_1 q_3 + r_2 q_0 - r_3 q_1 \\ r_0 q_3 - r_1 q_2 + r_2 q_1 + r_3 q_0 \end{bmatrix} \tag{4}$$

The inclination of the sensor is estimated using the acceleration expressed in the sensor frame $\boldsymbol{a_s}(t)$ (compare *Supplementary Figure 1*). The signal is assumed to have no movement component at FF event. Rotation angles Φ_{FF} around the anteroposterior axis (Y) and Ψ_{FF} around the lateral axis (Z) are calculated as:

$$\tan(\Phi_{\mathrm{FF}}) = -\frac{a_z}{\sqrt{a_x^2 + a_y^2}} \quad \text{and} \quad \tan(\Psi_{\mathrm{FF}}) = -\frac{a_y}{a_x} \tag{5}$$

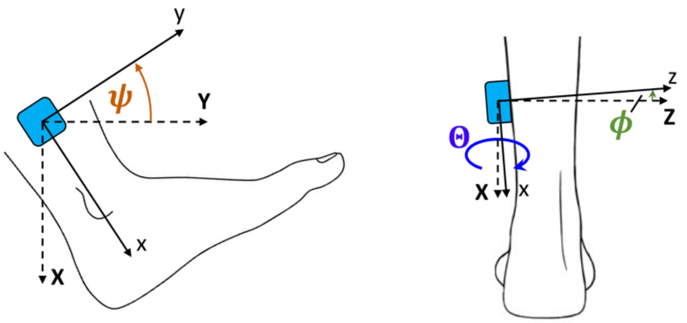

***Supplementary Figure 1**: Euler Angles in sensor tilting for transformation between sensor (xyz) and global frame (XYZ).*

The rotation Θ around the vertical (X) axis is not needed at this point since the YZ-plane movement is analyzed as a 2D vector. By using equation (3) with the two Euler angles Φ_{FF} and Ψ_{FF} at FF events, two rotation quaternions are defined as

$$\boldsymbol{q_y} = [\cos(\Phi_{\mathrm{FF}}/2), 0, \sin(\Phi_{\mathrm{FF}}/2), 0] \quad \text{and} \quad \boldsymbol{q_z} = [\cos(\Psi_{\mathrm{FF}}/2), 0, 0, \sin(\Psi_{\mathrm{FF}}/2)] \tag{6}$$

The two quaternions are combined using equation (4) to get the complete rotation at FF event:

$$\boldsymbol{q} = \boldsymbol{q_z} \otimes \boldsymbol{q_y} \tag{7}$$

The combined quaternion shows the orientation at FF event ($t = 0$). Due to changes of the foot orientation during the gait cycle, the initial quaternion at $t = 0$ needs to be updated at each sample within the stride ($t_{FF,i} < t < t_{FF,i+1}$). For updating, angular velocity $\boldsymbol{\omega}(t)$ is used:

$$\boldsymbol{q}(t) = \boldsymbol{q}(t-1) + \boldsymbol{q_{update}}(t), \quad (8)$$

$$\boldsymbol{q_{update}}(t) = 0.5 \cdot \frac{1}{f_{sampling}} \cdot \left(0, \boldsymbol{\omega}_x(t), \boldsymbol{\omega}_y(t), \boldsymbol{\omega}_z(t)\right)^T \quad (9)$$

The quaternion is normalized using the L_2 norm $\left(||\boldsymbol{q}(t)||_2 = \sqrt{q_0^2 + q_1^2 + q_2^2 + q_3^2}\right)$:

$$\boldsymbol{q}(t) = \frac{\boldsymbol{q}(t)}{||\boldsymbol{q}(t)||_2} \quad (10)$$

This final rotation quaternion represents the transformation from the sensor to global world frame and can be expressed as a rotation matrix:

$$R_{WS}(\boldsymbol{q}(t)) = \begin{bmatrix} 1 - 2(q_2^2 + q_3^2) & 2(q_1 q_2 - q_3 q_0) & 2(q_1 q_3 - q_2 q_0) \\ 2(q_1 q_2 - q_3 q_0) & 1 - 2(q_1^2 + q_3^2) & 2(q_2 q_3 - q_1 q_0) \\ 2(q_1 q_3 - q_2 q_0) & 2(q_2 q_3 - q_1 q_0) & 1 - 2(q_2^2 + q_2^2) \end{bmatrix} \quad (11)$$

4 Estimation of the 15 gait parameters

All 15 gait parameters are calculated individually for every gait cycle i. All temporal gait parameters are calculated from the gait events toe off (TO), maximal angular velocity (MAX), heel strike (HS) and foot flat (FF). All spatial gait parameter estimation methods use global velocity $\boldsymbol{v}(t)$ and position data $\boldsymbol{p}(t)$ combined with the gait events TO, MAX, HS and FF.

4.1 Spatial

1) **Stride length** (SL): The displacement of the foot is calculated in Y- and Z-direction between two FF events:

$$SL(i) = \sqrt{\left(\boldsymbol{p}_Y(t_{FF,i+1}) - \boldsymbol{p}_Y(t_{FF,i})\right)^2 + \left(\boldsymbol{p}_Z(t_{FF,i+1}) - \boldsymbol{p}_Z(t_{FF,i})\right)^2} \quad (12)$$

2) **Maximal foot clearance** (FC_{max}): We use the maximal relative sensor movement in vertical direction:

$$FC_{max}(i) = \max\{\boldsymbol{p}_{X,i}\} \quad (13)$$

3) **Gait velocity** (V_{Gait}): Gait velocity is calculated as the mean of $\boldsymbol{v}(t)$ in Y- and Z-direction during the gait cycle i:

$$V_{Gait}(i) = \frac{1}{n}\sum_{j=1}^{n} \sqrt{\boldsymbol{v}_{Y,i}(j)^2 + \boldsymbol{v}_{Z,i}(j)^2} \text{, where } n = \text{ number of samples for gait cycle } i \quad (14)$$

4) **Foot outward rotation** (Θ): The foot outward rotation can only be measured indirectly since an absolute reference of vertical angle Θ is missing with the IMU analysis. Therefore, the outward rotation is estimated from the ratio of foot displacement $\boldsymbol{d}$ in Z- and Y-direction:

$$\Theta(\mathrm{i}) = \arctan\left(\frac{\left|\boldsymbol{p}_Z\left(t_{FF,i+1}\right) - \boldsymbol{p}_Z\left(t_{FF,i}\right)\right|}{\left|\boldsymbol{p}_Y\left(t_{FF,i+1}\right) - \boldsymbol{p}_Y\left(t_{FF,i}\right)\right|}\right) = \arctan\left(\frac{d_Z}{d_Y}\right) \tag{15}$$

5) **Step width** (SW): Step width is approximated by observing the tilting angle Φ and match it to the width between the feet using the reference line obtained by the calibration measurement:

$$SW(i) = w \cdot \Phi\left(t_{FF,i}\right) + c \tag{16}$$

6) **Steps per 180° turn** ($n_{StepsTurning}$): The developed step detection algorithm is not robust for turning since the typical shape of the detection signal ω_z is varying much more from step to step than in continuous walking. Therefore, the number of steps during turning is evaluated by a simple maxima search in $\omega_x + \omega_z$ (without DTW). All turning sequences where the leg is reported to be horizontal are considered to estimate the steps during turning. The other sequences are discarded. The parameter $n_{StepsTurning}$ is normalized to 180° turning sequences:

$$n_{StepsTurning}(j) = \frac{n_{\mathrm{StepsInTurningSequence}}}{\mathrm{Turningangle}} \cdot 180° \quad , \quad \text{where } j = \text{ turning sequence} \tag{17}$$

7) **Arm swing amplitude** ($A_{swing,arm}$): The parameter captures the maximal angular velocity in the horizontal plane (in y- and z-direction):

$$A_{swing,arm}(i) = \max\left\{\left|\sqrt{\boldsymbol{\omega}_{arm,y}(t)^2 + \boldsymbol{\omega}_{arm,z}(t)^2}\right|\right\} \quad , \quad \text{where } t = t_{HS,i}, \ldots, t_{HS,i+1} \tag{18}$$

8) **Travelled arm distance** ($dist_{arm}$): Horizontal arm acceleration (in lateral and anteroposterior direction) is integrated twice between the two low motion times of the arm swing $t_{LM,arm}$ occurring between two steps . By observation, HS was found to be the event closest to $t_{LM,arm}$. Therefore, it is searched in a window around the HS event:

$$t_{LM,arm,i} = \mathrm{argmin}_{\mathrm{t} \in t_{HS,i}-0.3,\ldots,t_{HS,i}+0.3s}\left\{\left|\sqrt{\boldsymbol{\omega}_{arm,y}(t)^2 + \boldsymbol{\omega}_{arm,z}(t)^2}\right|\right\} \tag{19}$$

$\boldsymbol{a}_{arm}(t)$ is integrated between $t_{LM,arm,i}$ and $t_{LM,arm,i+1}$ for every gait cycle i and then dedrifted as described in Benoussaad et al. (2016) to get $\boldsymbol{v}_{arm}(t)$. $|\boldsymbol{v}_{arm}(t)|$ is integrated between $t_{LM,arm,i}$ and $t_{LM,arm,i+1}$ to get the relative travelled arm distances $\boldsymbol{d}_{arm}(i)$. The parameter $dist_{arm}(i)$ is obtained, by considering $d_{arm}(i)$ in the horizontal plane:

$$dist_{arm}(i) = \sqrt{d_{arm,Y}(i)^2 + d_{arm,Z}(i)^2} \tag{20}$$

4.2 Temporal

9) **Cycle time** (T_{cycle}): Is defined as the duration of one step, measured between two HS events:

$$T_{cycle}(i) = t_{HS,i+1} - t_{HS,i} \tag{21}$$

10) **Cycle time deviation** ($dev\{T_{cycle}\}$): Cycle time deviation is the normalized difference of the actual step duration to the mean of all performed steps:

$$dev\{T_{cycle}(i)\} = \frac{\left|T_{cycle}(i) - \bar{T}_{cycle}\right|}{\bar{T}_{cycle}}, \tag{22}$$

where $\bar{T}_{cycle} = \frac{1}{m}\sum_{i=1}^{m} T_{cycle}(i)$ and $m =$ number of steps

11) **Cadence** (n_{cycle}): The inverted value of T_{cycle} is the cadence, indicating the number of steps per second:

$$n_{cycle}(i) = \frac{1}{T_{cycle}(i)} \tag{23}$$

12) **Stance phase** (P_{stance}): The stance phase is characterized as ground contact of at least one foot point and is normalized to one gait cycle:

$$P_{stance}(i) = \frac{T_{stance}(i)}{T_{cycle}(i)}\text{, where } T_{stance}(i) = t_{TO,i} - t_{HS,i} \tag{24}$$

13) **Swing phase** (P_{swing}): The swing phase is characterized as no ground contact of the foot and is normalized to one gait cycle:

$$P_{swing}(i) = \frac{T_{swing}(i)}{T_{cycle}(i)}\text{, where } T_{swing}(i) = t_{HS,i+1} - t_{TO,i} \tag{25}$$

14) **Double limb support phase** (P_{DL}): The double limb support phase is present when both feet are in contact with the ground and is normalized to one gait cycle:

$$P_{DL}(i) = \frac{T_{DL}(i)}{T_{cycle}(i)}, \tag{26}$$

where $T_{DL}(i) = t_{TO,left,i} - t_{HS,right,i} + t_{TO,right,i} - t_{HS,left,i}$

15) **Stance to swing ratio** ($R_{StanceToSwing}$): To capture the relative behavior of stance and swing period, their ratio is monitored:

$$R_{StanceToSwing}(i) = \frac{T_{stance}(i)}{T_{swing}(i)} \tag{27}$$

The temporal parameters are depicted in *Supplementary Figure 2.*

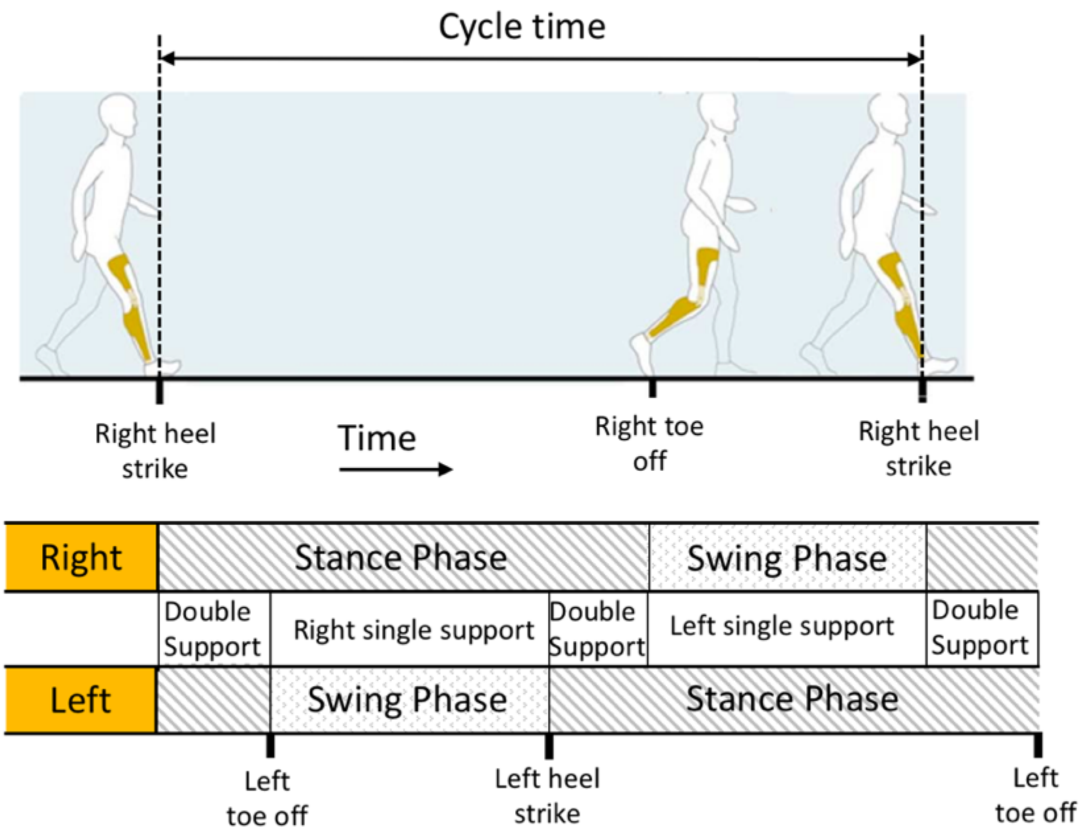

Supplementary Figure 2*: Timing of single and double support during a single gait cycle from right heel strike to the next right heel strike (adapted from (Qi et al., 2016) and (Whittle, 1991)).*

5 Validation measurement with Vicon motion tracking system

The used Vicon motion analysis system (Oxford Metrics Group, UK) consists of 10 ‘‘Vicon mx t160’’cameras with 16 Megapixels resolution at a sampling frequency of 200 Hz. Every marker is tracked with high accuracy throughout the procedure. A mean absolute system error less than 2 mm is reported under dynamic conditions (Merriaux et al., 2017). The marker placement and the test environment are depicted in *Supplementary Figure 3*.

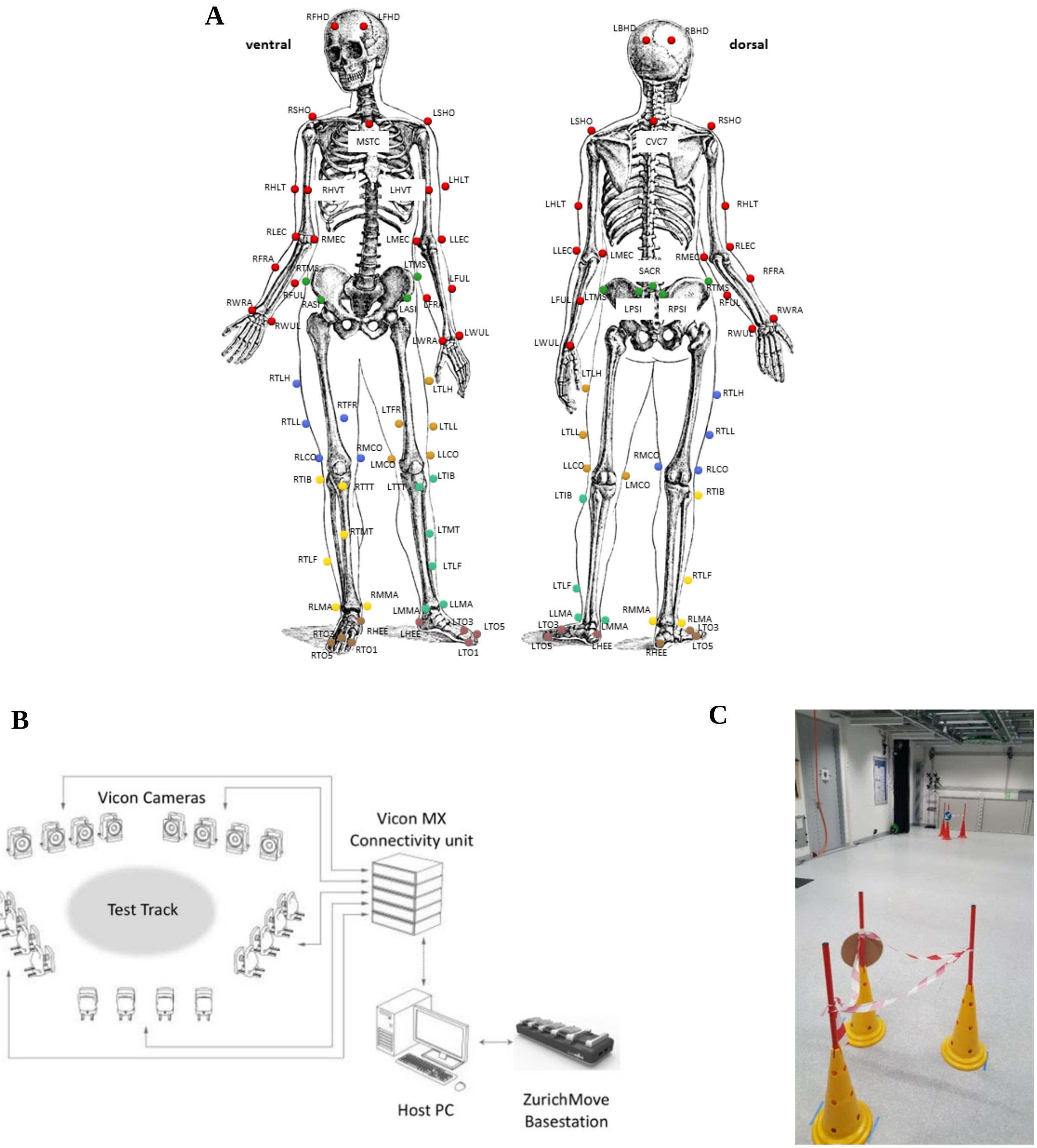

***Supplementary Figure 3**: (**A**): Marker placement on the body for gait parameter estimation with the Vicon system. (**B**): Test setup for the Vicon validation measurement showing the communication flow between Vicon cameras, Vicon connectivity unit, host PC and the ZurichMOVE base station. (adapted from Vicon User Guide (2017)) (**C**): The walked test track in the shape of an eight around cones.*

6 Gender comparison gait parameters

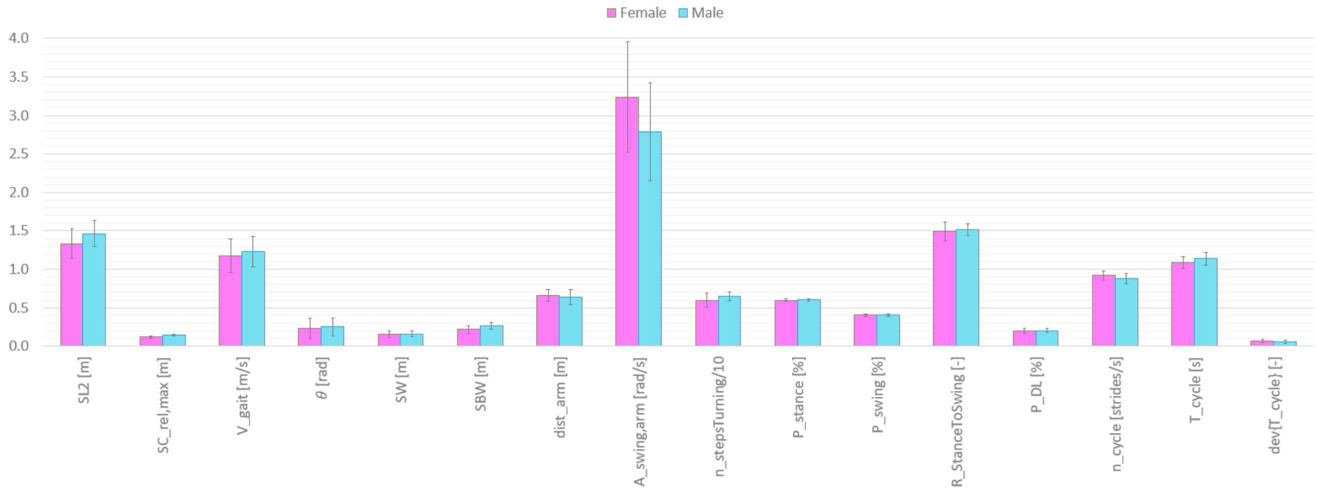

***Supplementary Figure 4**: Comparison between the gait parameters in the non-controlled home environment for female (n=20) and male subjects (n=20).*

References

Benoussaad, M., Sijobert, B., Mombaur, K., and Azevedo Coste, C. (2016). Robust foot clearance estimation based on the integration of foot-mounted IMU acceleration data. *Sensors* 16, 12.

Hannink, J., Ollenschläger, M., Kluge, F., Roth, N., Klucken, J., and Eskofier, B. M. (2017). Benchmarking foot trajectory estimation methods for mobile gait analysis. *Sensors (Switzerland)* 17. doi:10.3390/s17091940.

Merriaux, P., Dupuis, Y., Boutteau, R., Vasseur, P., and Savatier, X. (2017). A study of vicon system positioning performance. *Sensors* 17, 1591.

Müller, M. (2007). *Information retrieval for music and motion*. Springer.

Qi, Y., Soh, C. B., Gunawan, E., Low, K. S., and Thomas, R. (2016). Assessment of foot trajectory for human gait phase detection using wireless ultrasonic sensor network. *IEEE Trans. Neural Syst. Rehabil. Eng.* 24, 88–97. doi:10.1109/TNSRE.2015.2409123.

Whittle, M. W. (1991). *Gait Analysis: An Introduction.* Oxford: Butterworth-Heinemann.
